# Cortical Lewy body injections induce long-distance pathogenic alterations in the non-human primate brain

**DOI:** 10.1038/s41531-023-00579-w

**Published:** 2023-09-19

**Authors:** Margaux Teil, Sandra Dovero, Mathieu Bourdenx, Marie-Laure Arotcarena, Morgane Darricau, Gregory Porras, Marie-Laure Thiolat, Inés Trigo-Damas, Celine Perier, Cristina Estrada, Nuria Garcia-Carrillo, María Trinidad Herrero, Miquel Vila, José A. Obeso, Erwan Bezard, Benjamin Dehay

**Affiliations:** 1 Univ. Bordeaux, CNRS, IMN, UMR 5293, F-33000 Bordeaux, France; 2 HM CINAC, HM Puerta del Sur, Fundación HM Hospitales and CIBERNED and CEU-San Pablo University Madrid, E-28938 Mostoles, Spain; 3 Center for Networked Biomedical Research on Neurodegenerative Diseases (CIBERNED), Instituto Carlos III, Madrid, Spain; 4 Neurodegenerative Diseases Research Group, Vall d’Hebron Research Institute (VHIR), Barcelona, Spain; 5 Clinical and Experimental Neuroscience Unit, School of Medicine, Biomedical Research Institute of Murcia (IMIB), University of Murcia, Campus Mare Nostrum, 30100 Murcia, Spain; 6 Institute of Research on Aging (IUIE), School of Medicine, University of Murcia, 30100 Murcia, Spain; 7 Centro Experimental en Investigaciones Biomédica (CEIB), Universidad de Murcia, Murcia, Spain; 8 Department of Biochemistry and Molecular Biology, Autonomous University of Barcelona (UAB), Barcelona, Spain; 9 Catalan Institution for Research and Advanced Studies (ICREA), Barcelona, Spain; 10 Aligning Science Across Parkinson’s (ASAP) Collaborative Research Network, Chevy Chase, MD 20815 USA; 11 CEU, San Pablo University Madrid, E-28938 Mostoles, Spain 2 HM CINAC, HM Puerta del Sur and CIBERNED and CEU-San Pablo University Madrid, E-, 28938 Mostoles, Spain; 12 Present Address: UK Dementia Research Institute, University College London, London, WC1E 6BT UK

**Keywords:** Parkinson's disease, Neurodegeneration

## Abstract

Aggregation of α-synuclein (α-syn) is the cornerstone of neurodegenerative diseases termed synucleinopathies, which include Parkinson’s Disease (PD), Dementia with Lewy Bodies (DLB), and Multiple System Atrophy (MSA). These synucleinopathies are characterized by the deposit of aggregated α-syn in intracellular inclusions observable in neurons and glial cells. In PD and DLB, these aggregates, predominantly located in neurons, are called Lewy Bodies (LBs). These LBs are one of the pathological hallmarks of PD and DLB, alongside dopaminergic neuron loss in the substantia nigra. Previous studies have demonstrated the ability of PD patient-derived LB fractions to induce nigrostriatal neurodegeneration and α-syn pathology when injected into the striatum or the enteric nervous system of non-human primates. Here, we report the pathological consequences of injecting these LB fractions into the cortex of non-human primates. To this end, we inoculated mesencephalic PD patient-derived LB fractions into the prefrontal cortex of baboon monkeys terminated one year later. Extensive analyses were performed to evaluate pathological markers known to be affected in LB pathologies. We first assessed the hypothesized presence of phosphorylated α-syn at S129 (pSyn) in the prefrontal cortices. Second, we quantified the neuronal, microglial, and astrocytic cell survival in the same cortices. Third, we characterized these cortical LB injections’ putative impact on the integrity of the nigrostriatal system. Overall, we observed pSyn accumulation around the injection site in the dorsal prefrontal cortex, in connected cortical regions, and further towards the striatum, suggesting α-syn pathological propagation. The pathology was also accompanied by neuronal loss in these prefrontal cortical regions and the caudate nucleus, without, however, loss of nigral dopamine neurons. In conclusion, this pilot study provides novel data demonstrating the toxicity of patient-derived extracts, their potential to propagate from the cortex to the striatum in non-human primates, and a possible primate model of DLB.

## Introduction

Synucleinopathies constitute a group of age-related diseases that include Parkinson’s Disease (PD), Dementia with Lewy Bodies (DLB), and Multiple System Atrophy (MSA). These diseases are neurodegenerative disorders in which patients manifest motor and non-motor symptoms^1,2^. Despite their impact on the aging population, there are no current curative or halting treatments for the progression of these diseases^3^. To help discover treatments, it is essential to understand the mechanisms behind developing these synucleinopathies.

Synucleinopathies are characterized by α-synuclein (α-syn)-positive intracytoplasmic inclusions in either neurons or oligodendrocytes, depending on the disease^4,5^. In the case of PD and DLB, these neuronal inclusions are termed Lewy Bodies (LBs), whereas MSA patients present glial inclusions termed glial cytoplasmic inclusions (GCIs)^6^. Despite their different locations within the brain, these inclusions are associated with neuronal loss. The α-syn protein, under physiological conditions, is present predominantly at the pre-synaptic terminals and has shown multiple functions, including vesicular fusion, microtubule formation, and endocytosis^7–10^. In pathological conditions, misfolded α-syn has been suggested to act as a prion-like protein supporting these synucleinopathies through its ability to aggregate, spread from cell to cell, and seed its pathological information to endogenous monomeric α-syn^11–16^. The PD staging describing the spread of Lewy pathology, hypothesized by Braak and colleagues^15^, is one of the explanations for the disease progression. Previous observations suggest that specific neuronal populations are more vulnerable to α-syn pathology than others^17^. Therefore, it remains unclear how this vulnerability affects disease progression.

Previous studies have demonstrated that the injection of different polymorphs of misfolded α-syn can induce lesions reminiscent of synucleinopathies, including the appearance of pathological phosphorylated α-syn, dopaminergic neuron loss, neuroinflammation, and other markers^18^. These injection-based models focus on increasing α-syn levels in the brain through various techniques, including viral vector-based injections, injections of either preformed synthetic α-syn fibrils, or patient-derived brain materials^19–23^. Most of these studies have focused on injections of these materials in the nigrostriatal pathway to better mimic the effects of α-syn on this key pathway critically affected in synucleinopathies. In addition, these studies validated the use of these modalities to replicate some aspects of PD and MSA. However, few studies have yet focused on injecting materials into brain cortical areas to understand the propagation and vulnerability of cortical neurons affected in the later stages of these diseases.

This study thus aimed to understand better cortical neurons’ vulnerability and the potential of cell-to-cell propagation starting in the dorsal prefrontal cortex (dPFC). To this end, we injected non-human primates into their dPFC with LB fractions derived from the mesencephalon of PD patients, prepared as previously reported^24,25^. One year after administration, we investigated the neurobiological consequences in various cortical regions, the striatum, and substantia nigra pars compacta (SNpc). For the first time, we demonstrate the ability of these LB fractions to induce α-syn accumulation and neuronal loss in cortical areas as well as in the striatum but not in the SNpc.

## Results

### Widespread prefrontal phosphorylated α-syn immunoreactivity

**Fig. 1:**
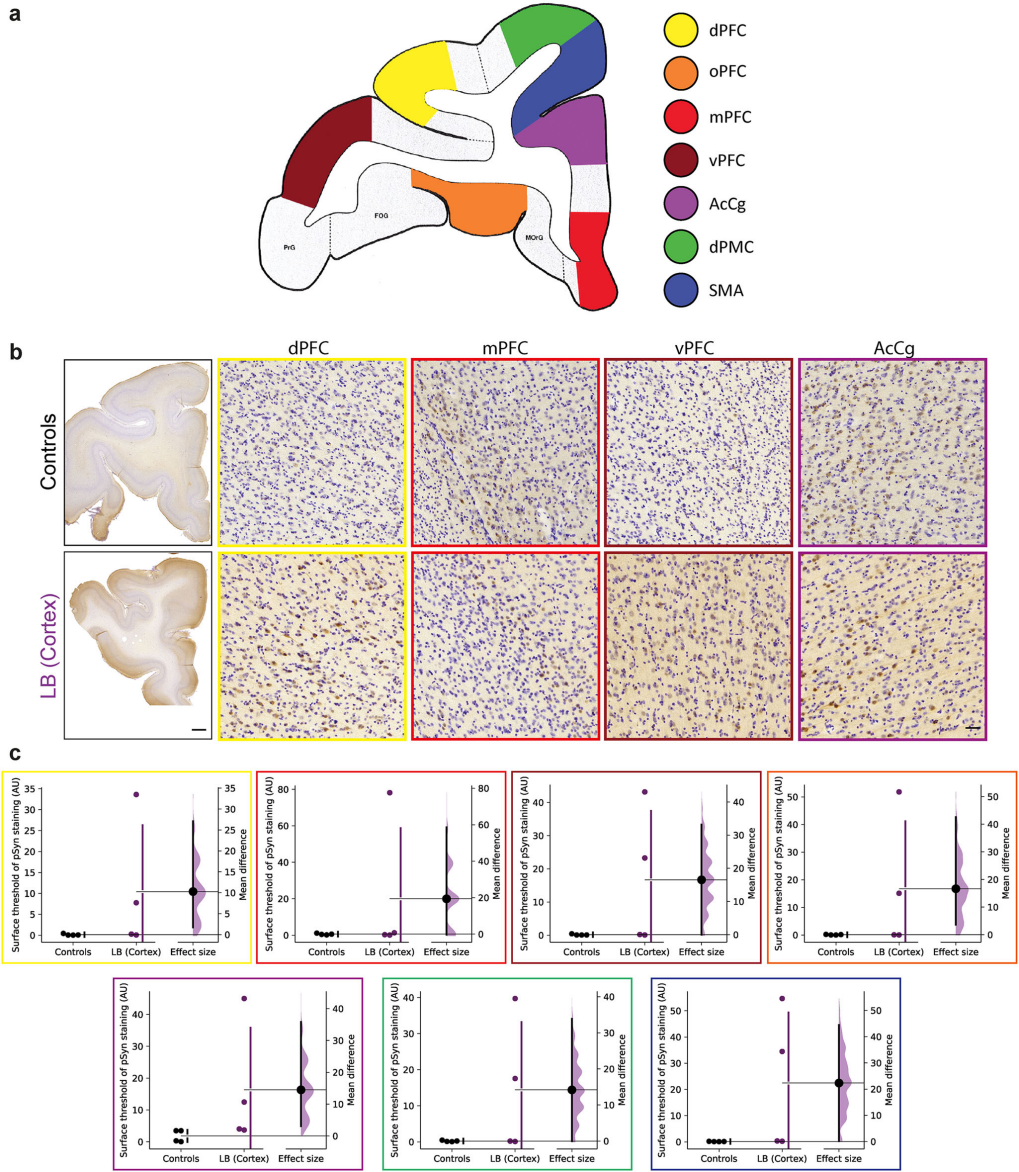
LB injections in the cortex of baboon monkeys induce an accumulation of cortical phosphorylated-α-synuclein. **a** (*left*) Schematic representation of prefrontal cortex level used as a reference for experiments (AC + 8) with colored areas analyzed. (*right*) Color-coded regions used for analysis in all experiments: yellow for dorsal prefrontal cortex (dPFC), orange for orbital prefrontal cortex (oPFC), red for medial prefrontal cortex (mPFC), brown for ventral prefrontal cortex (vPFC), purple for anterior cingulate cortex (AcCg), green for dorsal premotor cortex (dPMC), and blue for the supplementary motor area (SMA). **b** Representative images and **c** corresponding scatter plots of control and LB-injected monkeys with S129-phosphorylated α-synuclein staining in 7 different cortical regions: dPFC (yellow, *p* = 0.1213, *t* = 1.296), mPFC (red, *p* = 0.1782, *t* = 0.9987), vPFC (brown, *p* = 0.0813, *t* = 1.591), oPFC (orange, *p* = 0.1108, *t* = 1.364), AcCg (purple, *p* = 0.0955, *t* = 1.474), dPMC (green, *p* = 0.0739, *t* = 1.661), SMA (blue, *p* = 0.0909, *t* = 1.51). Scale bars: top 2 mm, bottom 50 μm. The horizontal line indicates the average value per group ± SD. The bootstrapped mean difference with 95% CI (error bar) is shown on the right side of each graph. Comparisons were made using unpaired *t*-tests.

We determined the consequences of one-year-old cortical LB injections in adult baboon monkeys’ dorsal prefrontal cortex (dPFC). To this end, we selected multiple inter-connected cortical regions to understand the effect of mesencephalic LB fractions on cortical neurons (Fig. 1a). As shown in mouse experiments, LBs are internalized in host cells by 24 h and disappear rapidly afterwards to trigger the endogenous pathological process^26,27^. Therefore, although the pathological α-syn species in LB contain phosphorylated α-syn at S129 (pSyn), we assume that after one year, the pSyn immunodetection in cortices reveals endogenous pSyn (Fig. 1b, c), as previously described^24,25,28^. LB injection in the dPFC increased pSyn staining in two out of four monkeys, showing trends in pSyn accumulation in LB-injected monkeys (Fig. 1c). This pSyn accumulation was observed in the dPFC, medial prefrontal cortex (mPFC), ventral prefrontal cortex (vPFC), orbital prefrontal cortex (oPFC), anterior cingulate cortex (AcCg), supplemental motor area (SMA), and dorsal premotor cortex (dPMC) (Fig. 1b, c).

**Fig. 2:**
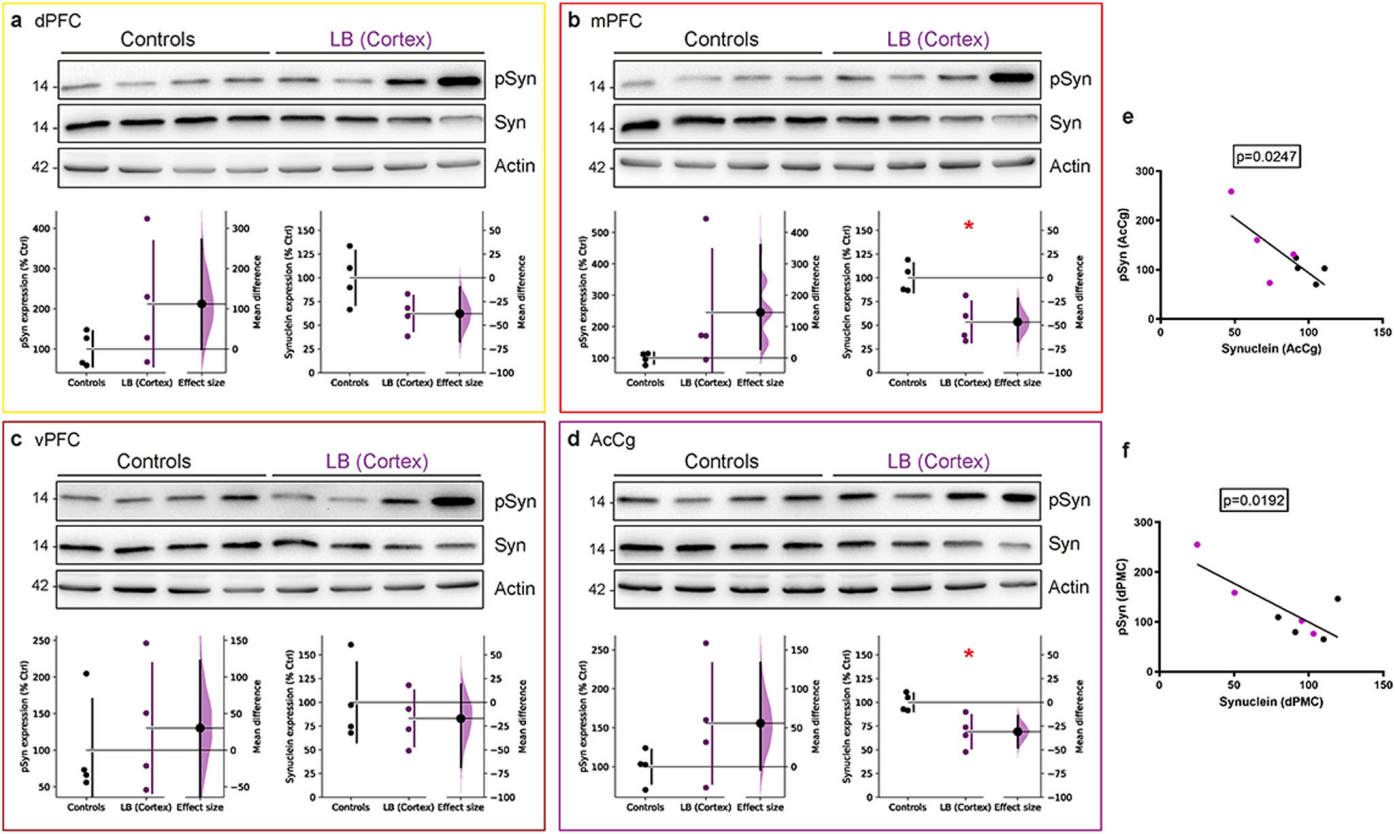
Total protein extracts of cortical regions reveal an accumulation of phosphorylated α-synuclein and a decrease in total α-synuclein proteins. **A**–**D** α-synuclein (Syn) and S129-phosphorylated α-synuclein (pSyn) immunoblot levels in the dorsal **(a)**, medial **(b)**, ventral prefrontal cortex **(c)** (dPFC, mPFC, vPFC respectively) and in the anterior cingulate cortex **(d)** (AcCg) in non-injected and LB-injected baboon monkeys (dPFC pSyn: *p* = 0.1077, *t* = 1.385; dPFC Syn: *p* = 0.0345, *t* = 2.213; mPFC pSyn: *p* = 0.1011, *t* = 1.432; mPFC Syn: *p* = 0.0066, *t* = 3.474; vPFC pSyn: *p* = 0.3055, *t* = 0.5364; vPFC Syn: *p* = 0.2661, *t* = 0.6627; AcCg pSyn: *p* = 0.1079, *t* = 1.384; AcCg Syn: *p* = 0.0105, *t* = 3.101). **e, f** Linear regression between α-syn expression and pSyn expression in the AcCg **(e)** (*p* = 0.0247, *F* = 8.865, *r*^*2*^ = 0.5964) and dorsal premotor cortex **(f)** (dPMC, *p* = 0.0192, *F* = 10.07, *r*^*2*^ = 0.6266). Each dot represents one monkey of the control (black) and LB-injected NHPs (purple). The horizontal line indicates the average value per group ± SD. The bootstrapped mean difference with 95% CI (error bar) is shown on the right side of each graph. Comparisons were made using unpaired *t*-tests, *p-value < 0.05.

Interestingly, these histological findings were corroborated by the increase of pSyn using biochemical investigations, shown by statistically positive correlations between immunohistochemistry and immunoblotting levels of pSyn in all investigated cortices (Fig. 2a–d, Supplementary Fig. 1). We also observed a parallel decrease in total α-syn expression levels (Fig. 2a–d). This decrease in α-syn expression levels was significant in the mPFC (Fig. 2b) and AcCg (Fig. 2d). This could be explained as the result of a change in the conformation of the α-syn protein to its pathological structure, therefore becoming undetected by the anti-total α-syn clone Syn211 antibody but not by the anti-p-S129 α-syn antibody, explaining an increase in one species associated with a decrease in the other. This is further confirmed by the negative correlations obtained in the AcCg and dPMC of α-syn and pSyn expression (Fig. 2e, f). Furthermore, using sequential extraction of Triton-X-soluble and -insoluble α-syn^29^, we did not detect any significant differences in Triton-X-insoluble monomeric, and high molecular weight of total α-syn and pSyn in dPFC, vPFC and AcCg (Supplementary Fig. 2). Unfortunately, we could not assess mPFC as a lack of remaining samples. Together, these results demonstrate that LB cortical injections induce an accumulation of pSyn in inter-connected cortical regions to the injection site, implying α-synuclein pathology propagation to prefrontal cortical areas connected to the injection location.

### Prefrontal neuronal loss

**Fig. 3:**
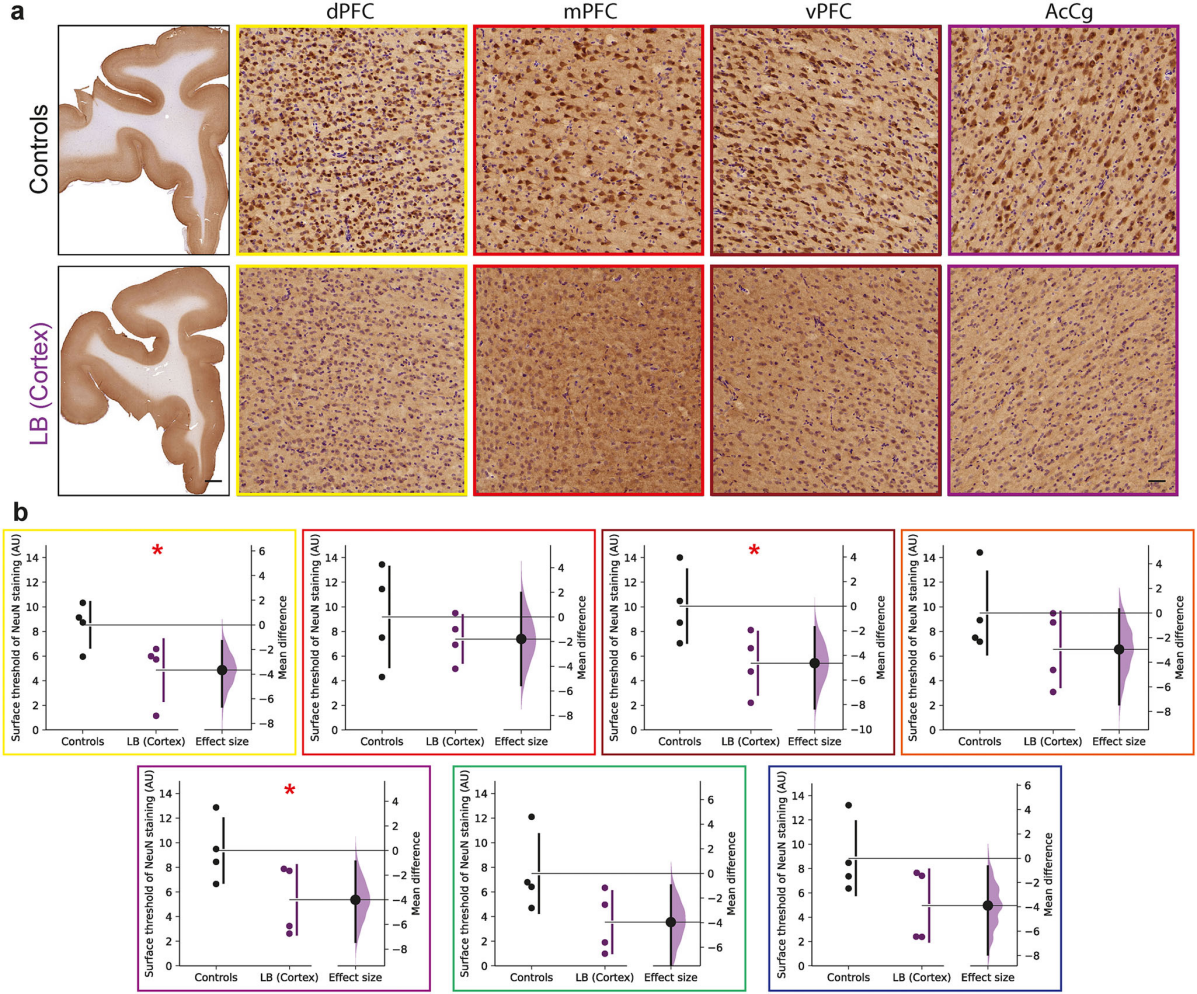
Intracortical LB injections induce loss of neurons in the prefrontal cortex after one year. Representative images **(a)** and corresponding scatter plots **(b)** of control and LB-injected monkeys with neuronal NeuN staining in 7 different cortical regions: dorsal prefrontal cortex (dPFC, yellow, *p* = 0.028, *t* = 2.364), medial prefrontal cortex (mPFC, red, *p* = 0.2296, *t* = 0.7908), ventral prefrontal cortex (vPFC, brown, *p* = 0.0281, *t* = 2.362), orbital prefrontal cortex (oPFC, orange, *p* = 0.1206, *t* = 1.3), anterior cingulate cortex (AcCg, purple, 0.0414, *t* = 2.079), dorsal premotor cortex (dPMC, green, *p* = 0.0502, *t* = 1.94), supplementary motor area (SMA, blue, *p* = 0.0581, *t* = 1.835). Scale bars: top 2 mm, bottom 50μm. The horizontal line indicates the average value per group ± SD. The bootstrapped mean difference with 95% CI (error bar) is shown on the right side of each graph. Comparisons were made using unpaired *t*-tests, *p-value < 0.05.

Given that pSyn immunoreactivity is associated with the loss of neurons in pathological studies^30–32^, we evaluated whether cortical LB injections could have similar effects in the PFC of baboon monkeys (Fig. 3). We measured the neuron-specific protein NeuN immunoreactivity in the same cortical regions (Fig. 3a)^33^. Analysis of NeuN staining indicated a loss of neurons in the dPFC, vPFC, and AcCg in cortical LB-injected monkeys compared to control monkeys (Fig. 3b). No significant changes were observed in the other cortical regions analyzed, most likely due to the small sample size, but trends were observed. This general neuronal loss in the injected site and connected cortical regions indicate neurotoxicity echoing the pSyn immunoreactivity.

### Prefrontal inflammation

**Fig. 4:**
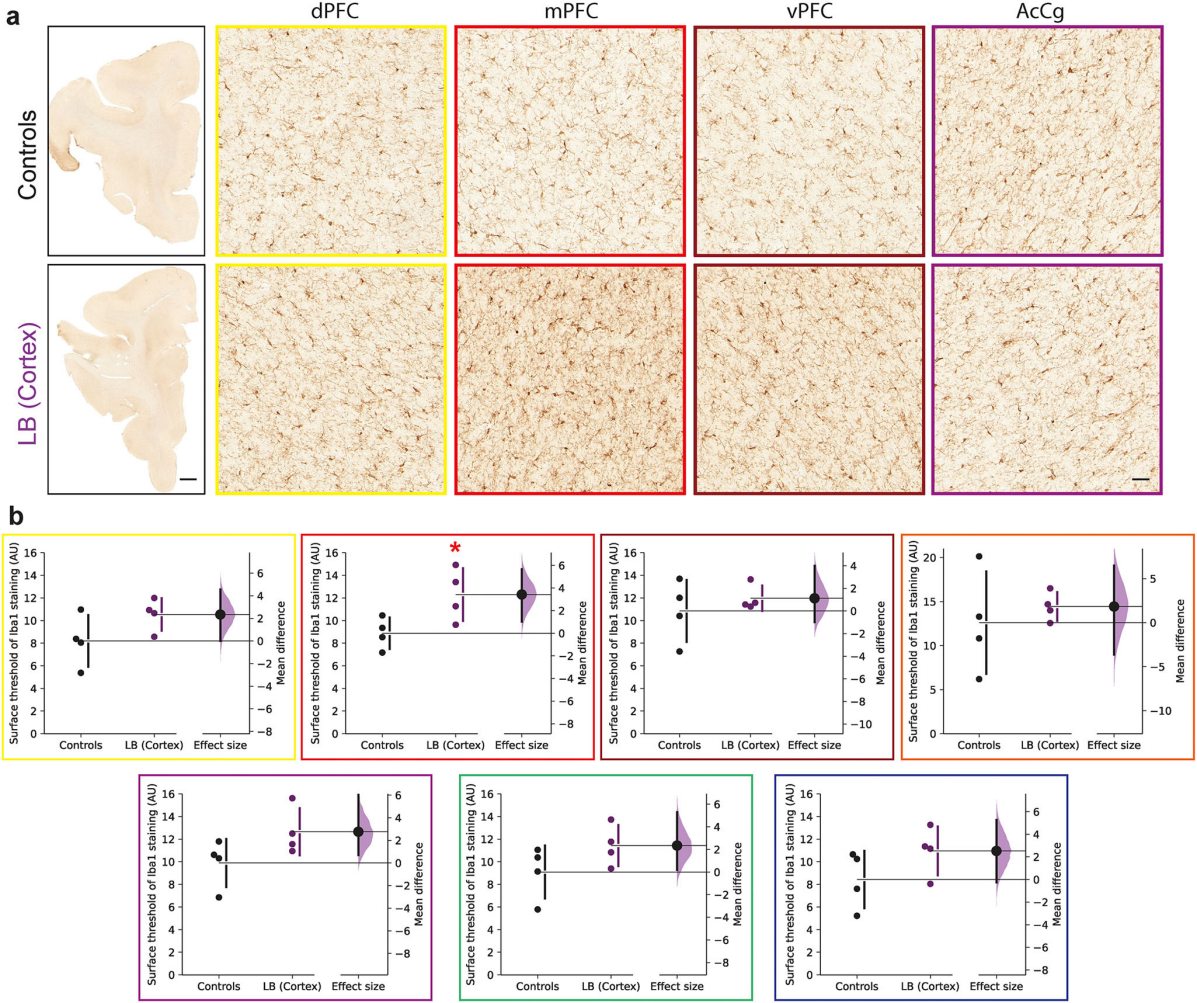
Microglia are increased in cortical LB-injected monkeys compared to controls. Representative images **(a)** and corresponding scatter plots **(b)** of control and LB-injected monkeys with microglial Iba1 staining in 7 different cortical regions: dorsal prefrontal cortex (dPFC, yellow, *p* = 0.0669, *t* = 1.733), medial prefrontal cortex (mPFC, red, *p* = 0.0223, *t* = 2.531), ventral prefrontal cortex (vPFC, brown, *p* = 0.2385, *t* = 0.7582), orbital prefrontal cortex (oPFC, orange, *p* = 0.2833, *t* = 0.606), anterior cingulate cortex (AcCg, purple, *p* = 0.0562, *t* = 1.858), dorsal premotor cortex (dPMC, green, *p* = 0.0824, *t* = 1.582), supplementary motor area (SMA, blue, *p* = 0.09, *t* = 1.518). Scale bars: top 2 mm, bottom 50 μm. The horizontal line indicates the average value per group ± SD. The bootstrapped mean difference with 95% CI (error bar) is shown on the right side of each graph. Comparisons were made using unpaired *t*-tests, *p-value < 0.05.

Knowing that pSyn immunoreactivity is also often associated with neuroinflammation, we next wanted to determine whether the LB injections resulted in changes in microglia or astroglia. We first assessed the potential microglial increased staining in the cortex and inter-connected cortical regions using Iba1 staining (Fig. 4a). In the mPFC, we observed a significant increase in microglial staining, accompanied by trends to increases in the other inter-connected cortical regions (Fig. 4b). To supplement this analysis, we quantified microglial network complexity in detail by fractal analysis. This method accurately depicts subtle changes in microglial morphology^34,35^. To this end, we isolated microglial cells from binarized images of Iba1 DAB-stained prefrontal sections (Fig. 4) and calculated individual values for classical shape descriptors (form factor and density). Outlined binary images were then used to calculate those quantitative parameters that provide a statistical index of complexity; hence indicative of the degree of the ramification of a microglial cell and inversely proportional to its degree of activation (Supplementary Fig. 3). We detected significant higher values in the density in LB-injected monkeys, indicating increased microglia network complexity in different cortical areas, confirming our increased staining in all cortical areas (i.e., dPFC, mPFC, vPFC, oPFC, AcGg, dPMC, SMA) (Supplementary Fig. 3). The form factor was slightly decreased only in LB-injected monkeys in dPFC, oPFC, and SMA, indicating a less rounded shape in microglia, while in the other cortical areas (i.e., mPFC, vPFC, AcGg, dPMC) no differences were observed. However, in the case of astroglia, we observed a significant decrease in GFAP staining in the dPFC and vPFC (Supplementary Fig. 4). Altogether, these results suggested that, in addition to pSyn accumulation and neuronal loss, cortical LB injections also induce an increase in microglia numbers and altered microglia morphology, indicating an ongoing inflammatory response throughout the PFC.

### Phosphorylated α-syn accumulates in the caudate nucleus but not in the putamen

**Fig. 5:**
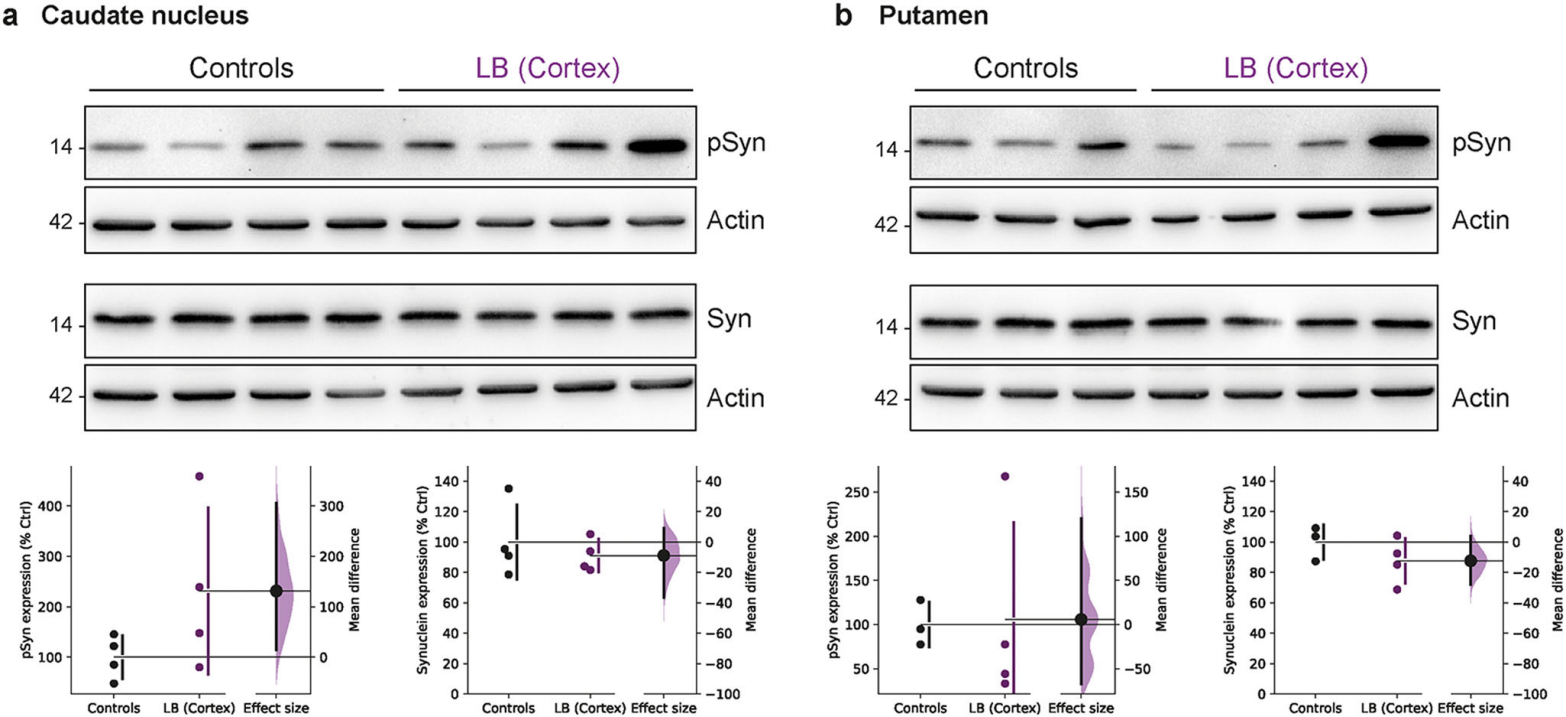
α-Synuclein accumulation is observed in the caudate nucleus of LB-injected baboon monkeys. **a, b** α-synuclein (Syn) and S129-phosphorylated α-synuclein (pSyn) immunoblot levels in the caudate nucleus **(a)** and putamen **(b)** in non-injected and LB-injected baboon monkeys (Caudate pSyn: *p* = 0.0871, *t* = 1.541; Caudate Syn: *p* = 0.2656, *t* = 0.6644; Putamen pSyn: *p* = 0.4677, *t* = 0.08519; Putamen Syn: *p* = 0.1425, *t* = 1.197). Each dot represents one monkey of the control (black) and LB-injected NHPs (purple). The horizontal line indicates the average value per group ± SD. The bootstrapped mean difference with 95% CI (error bar) is shown on the right side of each graph. Comparisons were made using unpaired *t*-tests.

Following our investigations in the PFC and associated cortical regions, we next attempted to examine the propagation of α-syn and its effects on connected downstream structures such as the striatum. We quantified the expression levels of both pSyn and α-syn in the caudate nucleus and the putamen by immunoblot analyses (Fig. 5). In the caudate nucleus, we observed a trend toward an increase in pSyn expression level with intracortical LB injections (*p* = 0.08) (Fig. 5a). However, no alterations in total α-syn expression level were observed. In the putamen, except for one monkey, no visible alterations were observed in pSyn or α-syn expression levels (Fig. 5b). This result could be related to frontal cortical areas preferentially projecting to the caudate nucleus. To assess whether these alterations in pSyn expression level between cortical regions and the striatum were associated, correlations were tested between the pSyn expression levels in the caudate nucleus and those of all previous regions analyzed (Supplementary Fig. 5). We found a positive correlation between the pSyn expression levels in the caudate nucleus and all other cortical and striatal areas observed. These results corroborate the hypothesis that these alterations in pSyn expressions are associated, and more significant changes would probably be kept later with the progression of pathology. Together, these results demonstrate that cortical injections of LB can induce a local increase in pSyn expression level and be transmitted to the caudate nucleus one-year post-inoculation.

### Striatal dopaminergic function is altered

**Fig. 6:**
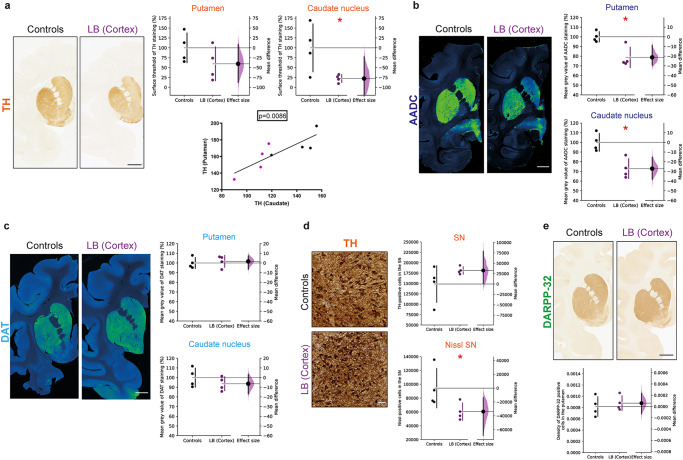
Alteration of dopaminergic staining in the striatum of LB-injected baboon monkeys, accompanied by a decrease in AADC staining. **a** Representative images of Control and LB-injected monkeys in the striatum, corresponding scatter plots of quantification of striatal immunostaining (Putamen: *p* = 0.1018, *t* = 1.426; Caudate: *p* = 0.022, *t* = 2.541) and linear regression between TH staining in the putamen and caudate nucleus (*p* = 0.0086, *F* = 14.75, *r*^*2*^ = 0.7108). **b, c** Representative images and quantification of AADC **(b)** and DAT **(c)** staining at a striatal level in control and LB-injected monkeys (AADC put: *p* = 0.0056, *t* = 3.606; AADC cd: *p* = 0.0037, *t* = 3.969; DAT pu: *p* = 0.3531, *t* = 0.3955; DAT cd: *p* = 0.1667, *t* = 1.052). **d** Representative images of Control and LB-injected monkeys in the substantia nigra (SN), corresponding scatter plots of quantification of nigral immunostaining (SNpc: *p* = 0.0989, *t* = 1.448; Nissl: *p* = 0.0362, *t* = 2.177). **e** Representative images and quantification of DARPP-32 staining at the striatal level in control and LB-injected monkeys (DARPP-32: *p* = 0.3018, *t* = 0.5478). Scale bars: top 5 mm, bottom 10 μm. Each dot represents one monkey of the control (black) and LB-injected NHPs (purple). The horizontal line indicates the average value per group ± SD. The bootstrapped mean difference with 95 % CI (error bar) is shown on the right side of each graph. Comparisons were made using unpaired *t*-tests, *p-value < 0.05.

Pathological α-syn increases were previously associated with dopaminergic terminal denervation, particularly after striatal or enteric nervous system administration of the same LB preparation^24,25^. Despite the subtle differences in pSyn levels in the caudate nucleus and the putamen, we wondered about putative dopaminergic terminal denervation in the striatum of cortical LB-injected monkeys (Fig. 6). Cortical LB injection induced a 75% loss of TH staining in the caudate nucleus. In contrast, no significant changes were observed in the putamen. However, we observed around 40% loss of TH staining (Fig. 6a). These alterations in striatal TH immunostaining correlated between the caudate nucleus and the putamen, indicating a delayed structure-dependent loss in the putamen (Fig. 6a), in line with the differential pSyn accumulation between the caudate nucleus and the putamen (Fig. 5). Further supporting the occurrence of ongoing striatal dopaminergic denervation, we also quantified AADC. This enzyme decarboxylates L-DOPA, and DAT, the dopamine transporter, levels. We observed a significant decrease in AADC, of 20–25%, in both the caudate nucleus and the putamen of LB-injected monkeys (Fig. 6b). However, the striatal expression of DAT remained unchanged between the LB-injected and control monkeys (Fig. 6c). Under physiological conditions, α-syn also controls dopamine storage in synaptic vesicles by interacting with the expression and activity of vesicular monoamine transporter 2 (VMAT2) in nigral neurons. Since VMAT2 is essential for reducing the harmful oxidative effects of DA metabolites in the cytosol, impaired DA sequestration in synaptic vesicles caused by abnormal α-syn may represent a pathogenic event in the degeneration of dopaminergic neurons^36^. We thus analyzed the striatal VMAT2 content and observed that cortical LB injection did not alter their protein levels (Supplementary Fig. 6), as shown between putamen or caudate tissue of controls and Incidental Lewy body disease cases^37^.

Considering the decrease in TH and AADC while having no loss of DAT or VMAT2, we conclude that the dopaminergic function must be altered (TH and AADC are both involved in dopamine production). In contrast, actual dopaminergic terminals and synaptic vesicle levels remain intact (stable DAT and VMAT2 levels). Further physiological explorations would be required to support the dysfunction of the dopaminergic transmission hypothesis.

### Lack of striatal and nigral degeneration

This previous conclusion is further corroborated by the lack of dopaminergic nigrostriatal neuronal loss, as evidenced by stereological counting of TH-positive neurons in the SNpc (Fig. 6d). The SNpc decrease in Nissl-positive cells in the cortical LB-injected monkeys suggests an alteration of non-dopaminergic nigral neurons. Previous striatal or enteric nervous system administration of the same LB preparation has led to significant dopaminergic nigrostriatal degeneration^24,25^. Cortical LB injection differs from those injection locations.

Although previous striatal or enteric nervous system administration of the same LB preparation had no impact upon medium spiny striatal neuron degeneration^24,25^, we reported such medium spiny neuron degeneration when monkeys were exposed to glial cytoplasmic inclusions derived from MSA patients^28^. We thus determined whether DARPP-32 staining, a marker of medium spiny neurons representing 90% of striatal neurons, was affected by cortical LB administration. DARPP-32 staining was not different between the experimental groups (Fig. 6e), suggesting that cortical LBs did not impact the survival of medium spiny neurons.

## Discussion

This pilot study demonstrated that, in non-human primates, mesencephalic LB injections lead to widespread prefrontal pSyn immunoreactivity, prefrontal neuronal loss, and prefrontal inflammation one year after intracortical administration. Such prefrontal cortex overt pathology was accompanied by pSyn accumulation in the caudate nucleus but not in the putamen, correlating with alteration of striatal dopaminergic function without, however, nigrostriatal denervation. These data suggest that mesencephalic LB affected the PFC over one year by propagating α-syn pathology (i.e., templating endogenous α-syn into pathologic forms) to the striatum, which would likely mature further in the long term.

We chose non-human primates as our preferred testbed to better understand synucleinopathies because of their similarities in brain anatomy and function with the human brain. Concerning the present work, an animal with a fully developed PFC that is reciprocally connected with the striatum and the presence of melanized dopamine nigral neurons was particularly important^38,39^. We recently contributed to understanding α-syn pathology propagation, a process underlying the spreading of pathological aggregates in the brain through the transfer of seeding-competent protein aggregates from one cell to another^40^, after stereotactic administration of the same (all 20–25 pg/µl of α-syn) patient-derived purified mesencephalic LB and striatal GCI fractions in the striatum of non-human primates^24,25,28^. These studies showed the potential for patient-derived α-syn aggregates to induce pathologies that start in the striatum and spread towards connected regions, i.e., SNpc and cortices^41^. By injecting these LB or GCI fractions, specific neuropathological features were observed depending on the location of these injections in the brain and the injected fraction. This was notably supported by the different profiles observed after striatal LB or GCI injections: LBs induced nigrostriatal denervation and cortical synuclein pathology while not killing medium spiny neurons^23,24^. GCIs also provoked nigrostriatal denervation, also associated with losing medium spiny neurons^28^.

Of particular relevance to the present work is the observation that the same LB preparation and quantity, when injected in the stomach wall, i.e., in close contact with the enteric nervous system (ENS), eventually leads to nigrostriatal degeneration and central nervous system pathology (though not cortical)^23^. However, what differs in the present study from the previous contributions is that the live phase was 12 months as opposed to 24 months of survival^24,25,28^. The reason for the neuropathological examination, performed 14 months post-injection, was to parallel our seminal paper^26^, knowing the results available at that time, as we knew that pathology was initiated from 9 months onwards, whether after injection in the striatum or in the SN^26^. Although the duration of the experiment can appear short compared to the course of the human disease, it is one of the most extended experiments ever reported in monkeys, as our previous study^26^ was 14 months after surgery, and the recent study from the Kordower lab^42^ was of 15 months. As we mentioned, this first and pilot experiment with this experimental design was to determine whether or not cortical SN-derived LB injection is associated with developing pSyn pathology. Knowing that ten years later, we can obviously and confidently go for a time course and afterwards time points, as we observe that the pathology maturation is still in its early stage, with caudate pSyn accumulation and TH fiber loss. With this in mind, the almost perfect linear regression we observed between the caudate nucleus and putamen TH density suggests that putaminal TH loss would become significant if a few extra months had been given to the process. PFC LB administration leads primarily to caudate TH fiber loss over the putamen TH fiber loss, which is compatible with the preferential PFC-caudate connections over the PFC-putamen connections^43,44^, and an overrepresentation of caudate versus putamen from the specifically targeted dPFC injection location. Pathology would therefore follow the density of projection that, in turn, would dictate the kinetic of pathology establishment and propagation.

Striatal injection of LBs and GCIs and gut injection of LBs induced nigrostriatal denervation^24,25,28^. The pathology maturation hypothesis explains at least partly why these dopaminergic neurons did not degenerate (yet). However, we report a significant loss of Nissl-positive cells in the SNpc. If confirmed, this finding challenges the specific vulnerability of dopaminergic neurons and fibers towards patient-derived α-syn aggregates since, after PFC administration of LBs, non-TH SNpc neurons degenerate while TH-positive neurons do not. Time seems more critical than connectivity as PFC is only two synapses away from SNpc through the striatum while the DA neurons are further away from the ENS neurons located in the stomach wall, which exposure to LBs leads two years later to significant DA neuronal loss^24^. Compensation is a known powerful feature of the nigrostriatal pathway, notably responsible for the late appearance of overt motor parkinsonian features despite the progressing extent of nigrostriatal denervation^45,46^. Compensation occurs in healthy dopamine neurons and the surviving neurons or the neurons whose terminals are compromised while the cell body remains present^45–49^. This echoes the hypothesis that the primary pathophysiological process relates to an “axonal dying back” of axons before the loss of neuronal bodies^50–52^. In our *princeps* study, we have also observed that the “dying-back” hypothesis applies to the LB experiments in non-human primates, as evidenced by more significant damage in the SNpc following striatal injections compared to nigral administration^26^. One-year post-injection would thus correspond to an intermediate state whereby dopaminergic fibers begin to be affected (caudate nucleus first versus putamen owing to the denser projections it receives from the PFC) without loss of dopamine neuron cell bodies in the SNpc.

Connectivity in the primate PFC has been extensively studied due to its implication in cognition, emotion, and behavior^53^. The primate PFC is divided into multiple subareas: dPFC, vPFC, mPFC and oPFC^53,54^. Globally, each PFC region is highly connected to its adjacent areas through short-distance connections, implicating high connectivity with the PFC, the PMC and the AcCg^54^. Given this high connectivity, we chose to quantify alterations in all PFC subregions, the AcCg and PMC. These many short-distance connections demonstrate the high propensity of α-syn to propagate to close-by cortical areas. This was seen in our data as pSyn staining was increased in PFC subregions and AcCg and PMC, with additional alterations of neuronal and microglial markers NeuN and Iba1, respectively, in these same close-by connected cortical areas.

A few mouse experiments, but none in non-human primates, have studied the consequences of cortical injection of α-syn material, either of patient-derived fractions, recombinant α-syn preformed fibrils (PFFs) or virus-driven α-syn-overexpression. These studies relied, for instance, upon using PFF cortical injections solely in the somatosensory cortex of wild-type mice, transgenic α-syn overexpressing mice^55^ or with concomitant PFF injections in the striatum^56^. These studies initiated the injections in cortical regions. They demonstrated the potential for seeding through likely connected cortical neurons after mPFC injections^57–60^, i.e., in a cortex closely related to the one we targeted in a non-human primate, led to pathology in the neighboring cortices^59,60^. These data showed the ability of aggregated or misfolded α-syn to seed from one cortex to another, with some striatal pathology reported at times^56,59^. Most of these studies, however, used large amounts of PFFs, i.e., in the microgram range as opposed to the picogram range of the brain-derived extracts^24,25,28,55,56^. Such cortical propagation is confirmed here with low quantities of aggregated α-syn in a non-human primate brain.

When assessing the effect of α-syn injections in the cortex, parallels between other synucleinopathies, particularly DLB, could be envisioned. Contrarily to PD patients, DLB patients present the formation of LBs diffusely in the cortical and limbic regions, primarily^61–63^. However, DLB also presents symptoms that encompass dementia, with neuropathological features such as the presence of amyloid-β (Aβ) plaques and tau tangles, similar to Alzheimer’s disease^64,65^. Neuropathologically, DLB presents cortical LBs and plaques, accompanied by neuronal loss and widespread cerebral atrophy^66^. In addition, it has been shown that morphologically, LBs can be divided into two categories, cortical LBs and LBs of the lower brainstem nuclei. Cortical LBs consist only of the dense core and are positive for α-synuclein. On the other hand, LBs from the lower brainstem nuclei possess the fibril halo^67,68^. These two types of LBs are also distinguished by their α-synuclein composition, with 64% of LBs in SNpc being positive for α-synuclein compared with 31% of cortical LBs^69^. Finally, LBs are not only present in PD but are a signature of a group of pathologies called dementia with Lewy bodies (DLB).

Here, our data demonstrate that prefrontal cortical LB injections can induce the pathological accumulation of pSyn and neuronal loss, similar to DLB patients^70^. However, no dementia-related disturbances were (yet) observed in these non-human primates at this time-point. In addition, creating an ideal model for DLB pathology would likely require co-injections of LB fractions in addition to fibrillar Aβ or with tau protein to recapitulate all aspects of pathology. In this case, one would hope to observe α-syn pathology accompanied by Aβ plaques, tau tangles, and cerebral atrophy.

However, this study has certain limitations, starting with the small cohort sizes with four animals per group, a number accepted in macaque research for both scientific and ethical reasons^71^. With two groups of four baboon monkeys, either LB-injected or non-injected, it is challenging to obtain homogenous results that allow giving definitive answers. Some endpoints do not reach statistical significance because of the small sample size. Hence the importance of the Gardner–Altman plots that focus upon the effect size of the difference, when any. For instance, in two LB-injected monkeys, we report an increase in pSyn, accompanied by neuronal loss both in the cortical regions and the striatum. Given the variabilities we can observe between non-human primates, it remains essential to consider these alterations. An additional question concerns the nature of the control arm in this exploratory study. At the outset of this large-scale study, we had to predetermine the experimental groups with a known number of animals and make choices regarding the control groups. Prior to our experiments with olive baboons^24,25,29^, only two studies reported the use of this species to model synucleinopathy and neurodegeneration^30,31^. We were therefore faced with a lack of basic parameters, for example, references for dopaminergic-related parameters.

The present study is the DLB component of a broad effort to explore the impact of PD- and MSA-derived α-syn aggregates in NHPs^21,24,25,29^. Non-injected animals were therefore used as controls for multiple extracts, thus avoiding the use of several additional control individuals in accordance with the 3 R principle. This choice was validated a posteriori by discovering that, in NHPs but not in mice, a small amount of singular small aggregates of PD-derived α-syn is as toxic as the large PD-derived amyloid fibrils present in LB^25^. With regard to this issue, we did not inoculate animals with α-syn fractions immunodepleted or denatured with formic acid, as we have previously shown that inoculation of immunodepleted α-syn fractions did not induce neurodegeneration or synuclein pathology^26^.

Another limitation is our reliance upon pSyn staining as reflecting pathological forms of α-syn. Following our recent opinion paper on the need to disclose the analysis of experimental synucleinopathies better, highlighting that the semantic and conceptual efforts should be made by scientists working with human tissues and experimental/animal material^72^, we acknowledge this limitation in the absence of true LB found in the NHP brains.

In conclusion, this pilot study demonstrated the ability of mesencephalic LB fractions to induce α-syn propagation to anatomically connected brain regions from the PFC and towards the caudate nucleus. This α-syn propagation was accompanied by neuronal loss and microglial activation, indicating a progressive induction of early stages of pathology. Potential next steps should, however, be expanded and confirmed using larger cohorts and could include the injection of cortical LB fractions to observe the varying cellular vulnerability and the potential for more pronounced pathological effects. These findings could lead to a better understanding of α-syn propagation and the role of the PFC in LB pathology.

## Methods

### Ethics statement

Experiments were performed following the European Union directive of September 22, 2010 (2010/63/EU) on protecting animals for scientific purposes. The Institutional Animal Care and Ethical Committee of Murcia University (Spain) approved non-human primate experiments under the license number REGA ES300305440012.

### Purification of LBs from human PD brains

LB purification was conducted as previously described^24–26,73^. The data reporting the complete characterization of α-syn used for the inoculations were not included in this study but in our previous publication^25^. The samples were obtained from brains collected in a Brain Donation Program of the Brain Bank “GIE Neuro-CEB” run by a consortium of Patients Associations: ARSEP (association for research on multiple sclerosis), CSC (cerebellar ataxias), France Alzheimer, and France Parkinson. The consents were signed by the patients themselves or their next kin in their name, following the French Bioethical Laws. The Brain Bank GIE Neuro-CEB (Bioresource Research Impact Factor number BB-0033–00011) has been declared at the Ministry of Higher Education and Research and has received approval to distribute samples (agreement AC-2013–1887). Human SNpc was dissected from fresh frozen postmortem midbrain samples from 5 patients with sporadic PD exhibiting conspicuous LB pathology on neuropathological examination (mean age at death: 67.5 ± 3.5 years; frozen postmortem interval: 17 ± 4 h; GIE Neuro-CEB BB-0033–00011). Tissue was homogenized in 9 vol (w/v) ice-cold MSE with protease inhibitor cocktail (Complete Mini; Boehringer Mannheim) with 12 strokes of a motor-driven glass/Teflon homogenizer. A sucrose step gradient was prepared for LB purification by overlaying 2.2 M with 1.4 M and finally with 1.2 M sucrose in volume ratios of 3.5:8:8 (v/v)^74^. The homogenate was layered on the gradient and centrifuged at 160,000xg for 3 h using an SW32.1 rotor (Beckman). Twenty-six fractions of 500 μl were collected from each gradient from the top (fraction 1) to bottom (fraction 26) and analyzed for the presence of α-syn aggregates by filter retardation assay, with 45 µl of each fraction deposited^26^. LB-containing fractions from PD patients were those between fractions 21 and 23. The amount of α-syn in the LB fractions was quantified using a human α-syn ELISA kit (#KHB0061; Invitrogen/Life Technologies, Carlsbad, CA, USA) and are reported in^25^. Quantification by ELISA indicated that the LB mix contained ∼24 pg of α-syn per microliter. Importantly, we used the same SN-derived LB fractions for all the experimental groups in this large-scale study, i.e., striatum-, ENS- and cortex-inoculated animals. Those SN-derived LB fractions have been fully characterized biochemically^25^ and demonstrated their ability to induce neurodegeneration and α-synuclein pathology after either striatal (reminiscent of our princeps article^26^) or gut injections. In all cases, samples were bath-sonicated for 5 min immediately before in vivo injections.

### Animals and stereotactic injections

Experiments were conducted as described at the research animal facility of the University of Murcia (Murcia, Spain)^24,25,28^. Adult female and male olive baboons (*n* = 8; *Papio papio*) ranging from 3 to 14 years of age were housed in two multi-male, multi-female exterior pens. Animals were fed fruits, vegetables, and dry food pellets twice daily before 9 am and after 5 pm. Water was available ad libitum. Allocation to experimental groups was randomized. Four baboons were used for LB injections, and four were untreated control animals. The control baboons were not injected but derived from the same large cohort colony (same initial breeders), age-matched with the injected animals, hosted in the same aviary and were run in parallel with the experimental group, i.e., cortex-inoculated animals^24,25,28^. Two reasons were behind this choice to keep a full age-matched and non-injected group. First, we faced a lack of baseline parameters in our hand, for instance, references of TH-positive cells, among others, as only two studies reported using baboons to model synucleinopathy and neurodegeneration in baboons^75,76^. Second, in the initial experimental design and based on the previous rodent studies, we did not expect degeneration in the noLB-injected animals^25^. However, since we observed that unexpected phenomenon, the reference group was used as a control group. Bilateral intracortical injections of LB fractions were performed at three levels of the dPFC [AP: +10; L: +21; DV: +1,2; +3,6; +6] under stereotactic guidance^24,25^. The total injected volume per hemisphere was 100 µl (3 injection sites of equal volumes at 3 µl/min at each location site). After each injection, the syringe was left in place for 10 min to prevent leakage along the needle track. Several parameters were monitored during the one-year study, including survival and clinical observations. At the end of the experiment, all baboons were terminated with pentobarbital overdose (150 mg/kg i.v.), followed by perfusion with room temperature 0.9% saline solution (containing 25 μl/ml heparin) following accepted European Veterinary Medical Association guidelines. Brains were removed quickly after death. Each brain was dissected along the midline, and each hemisphere was divided into three parts^24,25^. The left hemisphere was frozen immediately by immersion in a cold isopentane bath at −50 °C for at least 5 min and stored at −80 °C for further biochemistry investigations. The right hemisphere was fixed for one week in 10 vol/tissue of 4% paraformaldehyde at 4 °C, cryoprotected in two successive gradients of 20 then 30% sucrose in phosphate-buffered saline (PBS) (until they sunk) before being frozen by immersion in a cold isopentane bath (−50 °C) during at least 5 min and stored immediately at −80 °C until sectioning, as previously described^24,25,28^. No sample was excluded from the analysis in these studies.

### Histological analysis

#### Phosphorylated α-synuclein staining

Pathological handling of synuclein was assessed in monkeys with a mouse monoclonal antibody raised against human phosphorylated α-syn on S129 (clone11A5, Elan, 1:5000), as previously reported^25,26,28^. Briefly, selected sections at each animal’s prefrontal cortex (AC + 9) level were identified to distinguish each animal. Sections were incubated overnight at RT with the antibody mentioned above. The following day, the revelation was performed with anti-rabbit peroxidase EnVision system (DAKO Envision+ HRP, K400311–2) followed by 3,3′-diaminobenzidine (DAB, DAKO, K346811–2) incubation. Sections were then mounted on gelatinized slides, counterstained with 0.1% cresyl violet solution, dehydrated, and cover-slipped until further analysis. Slides were scanned with a high-resolution scanner (Pannoramic Scan II, 3DHISTECH Ltd, France) at x20 magnification and on five layers spaced by 1.4 µm each. Quantifications were estimated by immunostaining-positive surface quantification at regional levels with the Mercator software (IMASCOPE, France).

#### Neuronal loss

NeuN (Neuronal Nuclei) and cAMP-regulated neuronal phosphoprotein 32 kDa (DARPP-32) immunohistochemistry were performed to assess neuronal loss in the cortex and medium spiny neuron loss in the striatum, respectively. For NeuN staining, sections were selected in the prefrontal cortex and were incubated with mouse monoclonal NeuN antibody (Merck, MAB377, 1:1000). For DARPP-32 staining, sections were selected in the anterior striatum and incubated with DARPP-32 antibody (Merck, MAB4230, 1:500). Regardless of antibodies, sections were incubated overnight at RT. The next day, antibodies were revealed by the corresponding anti-species peroxidase EnVision (DAKO Envision+ HRP, K400111–2) secondary antibody, followed by DAB visualization. Free-floating NeuN stained sections were mounted on gelatin-coated slides, counterstained with 0.1% cresyl violet solution, dehydrated, and cover-slipped. For DARPP-32-stained sections, the slices received the same treatment but without cresyl violet counterstaining. Slides were scanned (Pannoramic Scan II, 3DHISTECH Ltd, France) and analyzed using the Mercator software (IMASCOPE, France). NeuN slides were analyzed using a surface threshold, where the baseline detection signal was determined and applied to all study slides. DARPP-32 slides were scanned using an Epson Expression 10000XL high-resolution scanner. Images were analyzed using ImageJ open-source software (version 1.53) to compare OD measuring the mean gray staining as previously described^28^.

#### Inflammation

Inflammatory processes in striatal sections were measured as previously described^24,25,28^ through GFAP/S100 (Merck, MAB360/Abcam, ab4066) and Iba1 (Abcam, ab5076) immunohistochemistry. Striatal sections were incubated overnight with a mix of mouse antibodies raised against GFAP and S100 for the astroglial staining (respective dilutions 1:2000 and 1:1000) and with a goat anti-Iba1 antibody for the microglial staining (dilution 1:1000). These signals were revealed with anti-species peroxidase EnVision system (DAKO) followed by DAB incubation. GFAP-S100 sections were mounted on slides, counterstained in 0.1% cresyl violet solution, dehydrated and cover-slipped. Sections stained by Iba1 were mounted on slides, dehydrated and cover-slipped. GFAP-S100 and Iba1 slides were scanned with a high-resolution scanner (Pannoramic Scan II, 3DHISTECH Ltd, France) at x20 magnification and on five layers spaced by 1.4 µm each. All quantifications were estimated by immunostaining-positive surface quantification at regional levels with the Mercator software (IMASCOPE, France).

#### Dopaminergic neurodegeneration assessment

To assess the effect of cortical LB injections on dopaminergic neurons and fibers, tyrosine hydroxylase (TH), AADC (Aromatic L-amino acid decarboxylase), and DAT (Dopamine Transporter) immunohistochemistry and quantifications were performed on striatal and SNpc sections as previously described^24,25,28^. For TH staining, sections were selected in the anterior striatum (pre-anterior commissure) and serial sections (1 to 12) of whole SNpc, AADC, and DAT in the anterior striatum (pre-anterior commissure). Sections were incubated with rabbit monoclonal TH antibody (Millipore, MAB318, 1:5000), rabbit polyclonal AADC antibody (Merck AB136, 1:1000), or rat monoclonal DAT antibody (Merck MAB369, 1:500) for one night at room temperature (RT) and revealed the next day with the corresponding peroxidase EnVision secondary antibody, followed by DAB visualization. SNpc sections were mounted on gelatin-coated slides, counterstained with 0.1% cresyl violet solution, dehydrated and cover-slipped. Striatal sections were mounted on gelatin-coated slides, dehydrated and cover-slipped. Striatal sections were analyzed by optical density (OD) in the caudate nucleus and putamen. The slides were scanned using Epson Expression 10000XL high-resolution scanner. Images were analyzed using ImageJ open-source software (version 1.53) to compare mean gray levels in the caudate nucleus and putamen. TH-positive neurons of the SNpc were counted by stereology blind about the experimental condition using a Leica DM6000B microscope coupled with the Mercator software (IMASCOPE, France). The SN was delineated for each slide, and dissector probes for stereological counting (100 × 80 µm spaced by 600 × 400 µm) were applied to the map obtained. Each TH-positive cell with the nucleus in the probe was counted following the stereological exclusion rules^24,25,28^. The total number of TH-positive neurons in the whole SN was then assessed per hemisphere using the optical fractionator method.

### Biochemical analysis

#### Total protein extraction

Tissue patches collected on 300 µm-thick cryostat-cut sections of caudate nucleus and putamen (*n* = 5–10 patches per structure and animal) were extracted on ice using 150–200 µl of RIPA buffer with a protease and phosphatase inhibitor cocktail as previously described^24,25,28^. Lysates were sonicated in a water bath for 10 min, then incubated for 30 min on ice before being centrifugated at 14,000 rpm for 15 min at 4 °C. Supernatants were collected, and the total amount of protein in the lysates was assessed by Bicinchoninic Acid (BCA) assay before storage at −80 °C.

Based on total protein concentrations from the BCA assays, aliquots of tissue lysates corresponding to known amounts of total protein per sample were prepared for each animal in Laemmli buffer (Tris–HCl 25 mM pH=6.8, Glycerol 7.5%, SDS 1%, DTT 250 mM, and Bromophenol Blue 0.05%) for immunoblotting experiments.

#### Sequential protein extraction

Tissue patches collected as above (n = 10 patches per structure and animal) were homogenized in Triton-X (TX) extraction buffer (50 mM Tris-base pH 7.6, 150 mM NaCl, 1% Triton-X-100, 2 mM EDTA) containing protease and phosphatase inhibitors as previously described^24,28,29^. The lysate was sonicated and then centrifuged (120,000 × *g* for 60 min at 4 °C), and the supernatant was collected (TX-soluble fraction). The pellet was then washed 3 times with 1 M PBS/1% TX, centrifuged (13,000 × *g* for 15 min) and re-suspended in SDS extraction buffer (50 mM Tris pH 7.6, 150 mM NaCl, 1% Triton-X-100, 0.5% Na- deoxycholate, 1% SDS), sonicated, and centrifuged (120,000 × *g* for 60 min at 4 °C) and the supernatant was collected (TX-insoluble fraction).

#### Immunoblotting

Western blots were run in all conditions using 20 μg of protein separated by SDS-PAGE and transferred to nitrocellulose membranes, as previously described^24,25^. Incubation of the primary antibodies was performed overnight at 4 °C with anti-phosphorylated-α-syn at Ser129 (1:5000, Abcam [EP1536Y], ab51253), total α-syn (1:1000, ThermoFisher [Syn211], 328100) and VMAT2 (1:1000, Abcam, ab191121). Anti-β-actin (1:10 000, Sigma, A5441) was used to control equal loading. A Super Signal West Chemiluminescent kit revealed appropriate secondary antibodies coupled to peroxidase (Immobilon Western, Chemiluminescent HRP substrate, Millipore). Chemiluminescence images were acquired using the ChemiDoc+XRS system measurement (BioRad). Signals per lane were quantified using ImageJ (version 1.53). A ratio (protein of interest normalized to β-Actin protein levels, then to Control values) of the signal on loading per animal was performed and used in statistical analyses.

### Microglial morphology

High-resolution z-stack images of Iba1-stained midbrain sections obtained using a high-resolution slide scanner (Pannoramic Scan II, 3DHISTECH Ltd, France) were opened in ImageJ, processed for image segmentation and fractal analysis by a semi-automated method. Briefly, z-stacks were converted to maximal projection images with the stack ‘sum’ function. Then, a region occupied by one microglial cell and its processes (but not processes from other cells) was delineated with the freehand tool, and an automatic local threshold (Otsu) was applied. Overlapping and out-of-focus cells were discarded. Box-counting fractal dimension was calculated on the resulting binary image with the FractalCount plugin for Fiji/ImageJ using default parameters. The area and perimeter of cells and their convex hulls were measured with ImageJ default tools to calculate shape descriptors. Fractal box-counting dimension was calculated for at least 50 cells per animal per region. Image analysis was performed in Fiji/ImageJ using custom scripts (available at https://github.com/SoriaFN).

### Statistical analysis

For all experiments, comparisons among means were performed using raw data using Student’s one-tailed unpaired t-test (GraphPad Prism 10.0, San Diego, CA). Correlations between variables were assessed with Spearman’s correlation analysis. Statistical significance was set at *p* < 0.05. The debate about the need to move beyond *p-value* is raging. Data must be analyzed further with estimation graphics^77^ emphasizing the effect size. Therefore, all data appear as estimation graphics called ‘Gardner–Altman plots’: on the left of each graph, data of controls and LB (Cortex) groups are presented as scatter plots showing the observed values along with above defined descriptive statistics (mean ± standard deviation). On the right of each graph is a contrast graph using the difference axis to display an effect size, which means difference. Horizontally aligned with the mean of the test group, the mean difference is indicated by the black circle. The black vertical line illustrates the 95% confidence interval (CI) of the mean difference. Given the observed data, the curve shows the resampled distribution of the effect size.

### Reporting summary

Further information on research design is available in the Nature Research Reporting Summary linked to this article.

### Supplementary information

Supplementary Information

SuppementaryTable 2

Reporting Summary

Supplementary Table 1

## Data Availability

The data supporting the findings of this study are provided in Supplementary Table 1, and the list of antibodies used in this study is provided in Supplementary Table 2. The data and material supporting the findings of this study are available from the corresponding authors on request.
