# Supplementary material for: Cortical Lewy body injections induce long-distance pathogenic alterations in the non-human primate brain
Source: NPJ Parkinsons Dis. 2023 Sep 19;9:135. doi: 10.1038/s41531-023-00579-w (PMC10509171; doi:10.1038/s41531-023-00579-w)
Supplement: Supplementary file 1 — Supplementary Information [file 41531_2023_579_MOESM1_ESM.docx]

**Supplementary Figures**


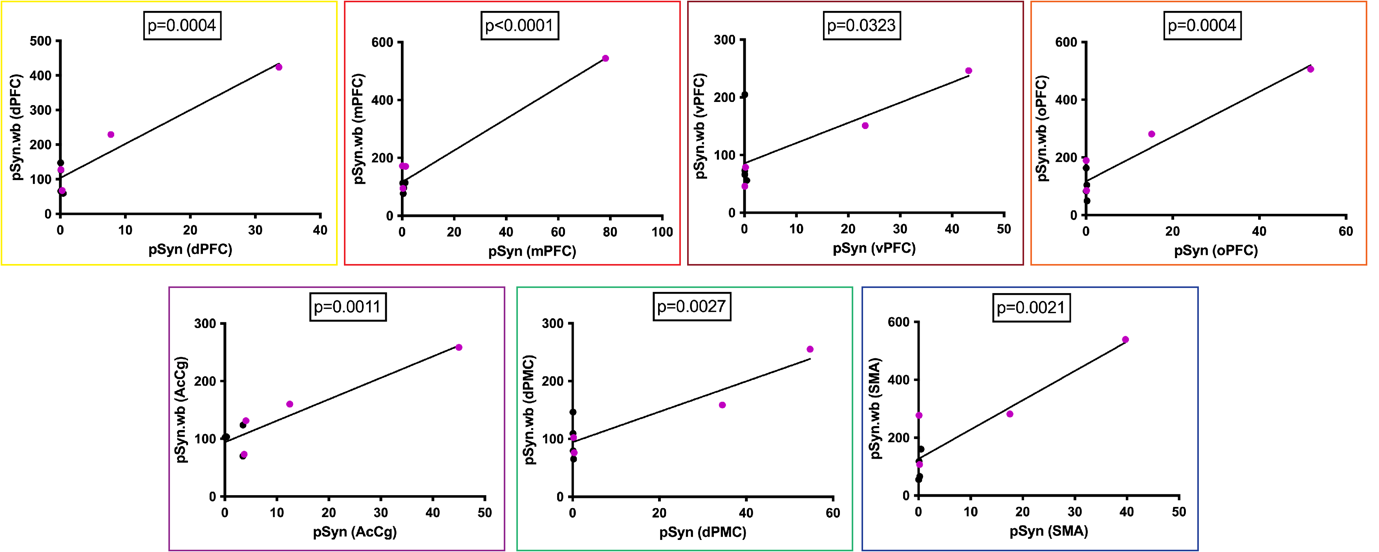


**Supplementary Fig. 1: Data from histological and biochemical techniques correlate in each region analysed for phosphorylated α-synuclein staining.** Linear regression between phosphorylated α-syn (pSyn) staining using histochemistry and immunoblotting in all 7 cortical areas examined: dorsal prefrontal cortex (dPFC, yellow, p=0.0004, F=51.58, r^2^ = 0.8958), medial prefrontal cortex (mPFC, red, p<0.0001, F=116.0, r^2^ = 0.9508), ventral prefrontal cortex (vPFC, brown, p=0.0323, F=7.686, r^2^ = 0.5616), orbital prefrontal cortex (oPFC, orange, p=0.0004, F=50.08, r^2^ = 0.893), anterior cingulate cortex (AcCg, purple, p=0.0011, F=33.86, r^2^ = 0.8495), dorsal premotor cortex (dPMC, green, p=0.0027, F=24.18, r^2^ = 0.8012), supplementary motor area (SMA, blue, p=0.0021, F=26.85, r^2^ = 0.8174). Each dot represents one monkey of the control (black) and LB-injected NHPs (purple).


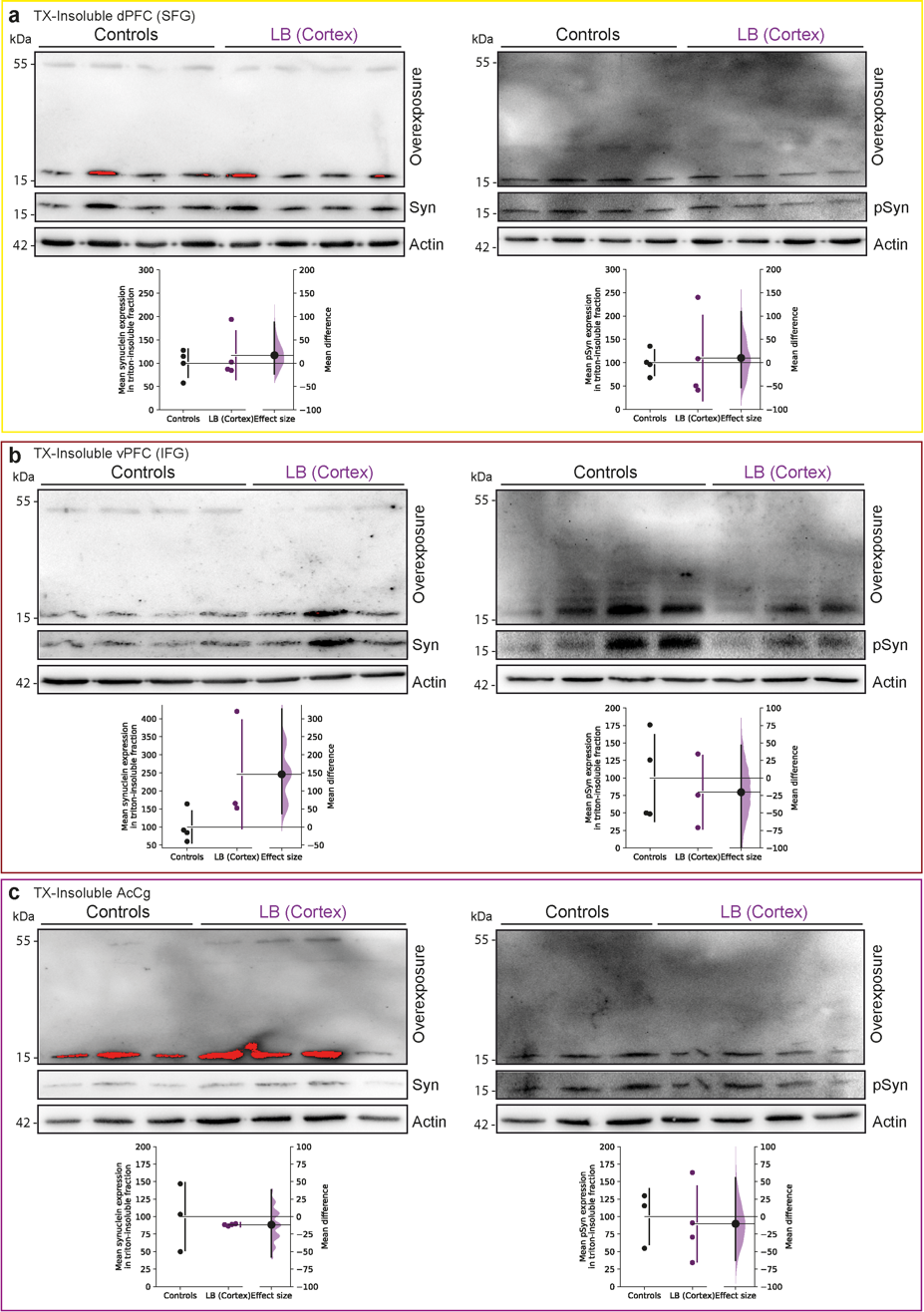


**Supplementary Fig. 2: Triton-Insoluble extracts of cortical regions reveal alterations in phosphorylated α-synuclein and total α-synuclein proteins.** (**a-c**) α-synuclein (Syn)(*left*) and S129-phosphorylated α-synuclein (pSyn)(*right*) immunoblot levels in the dorsal **(a)**, ventral **(b)** (dPFC and vPFC respectively) and in the anterior cingulate cortex **(c)** (AcCg) in non-injected and LB-injected baboon monkeys (dPFC Syn: p=0.2955, t=0.5675; dPFC pSyn: p=0.4205, t=0.2096; vPFC Syn: p=0.0593, t=1.881; vPFC pSyn: p=0.3337, t=0.4563; AcCg Syn: p=0.3209, t=0.4947; AcCg pSyn: p=0.3975, t=0.2741). Each dot represents one monkey of the control (black) and LB-injected NHPs (purple). The horizontal line indicates the average value per group ± SD. The bootstrapped mean difference with 95% CI (error bar) is shown on the right side of each graph. Comparisons were made using unpaired t-tests.


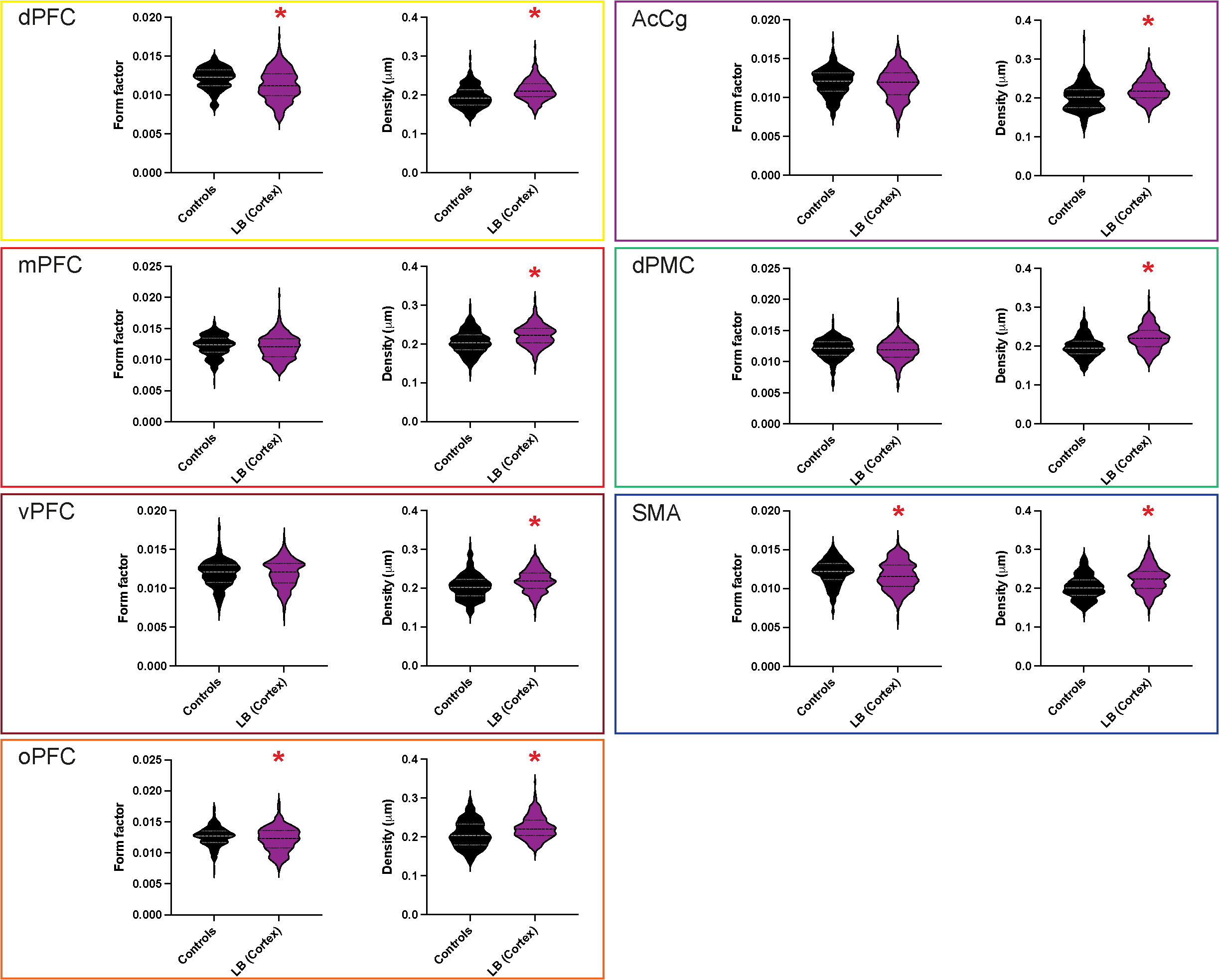


**Supplementary Fig. 3: Fractal dimension analysis of the PFC shows increased microglia network complexity in different cortical areas.** Scatter plots of two parameters displaying the form factor (*left*) and the density (*right*) in control and LB-injected monkeys in 7 different cortical regions: dorsal prefrontal cortex (dPFC, yellow, form factor: p<0.0001, t=5.301; density: p<0.0001, t=7.133), medial prefrontal cortex (mPFC, red, form factor: p=0.166, t=0.9712; density: p<0.0001, t=6.844), ventral prefrontal cortex (vPFC, brown, form factor: p=0.446, t=0.1358; density: p<0.0001, t=5.639), orbital prefrontal cortex (oPFC, orange, form factor: p=0.0118, t=2.27; density: p<0.0001, t=6.039), anterior cingulate cortex (AcCg, purple, form factor: p=0.1055, t=1.253; density: p<0.0001, t=7.1), dorsal premotor cortex (dPMC, green, form factor: p=0.1743, t=0.9382; density: p<0.0001, t=8.096), supplementary motor area (SMA, blue, form factor: p=0.0028, t=2.788; density: p<0.0001, t=6.766). The horizontal lines indicate medians and quartiles. Comparisons were made using unpaired t-tests, *p-value<0.05.


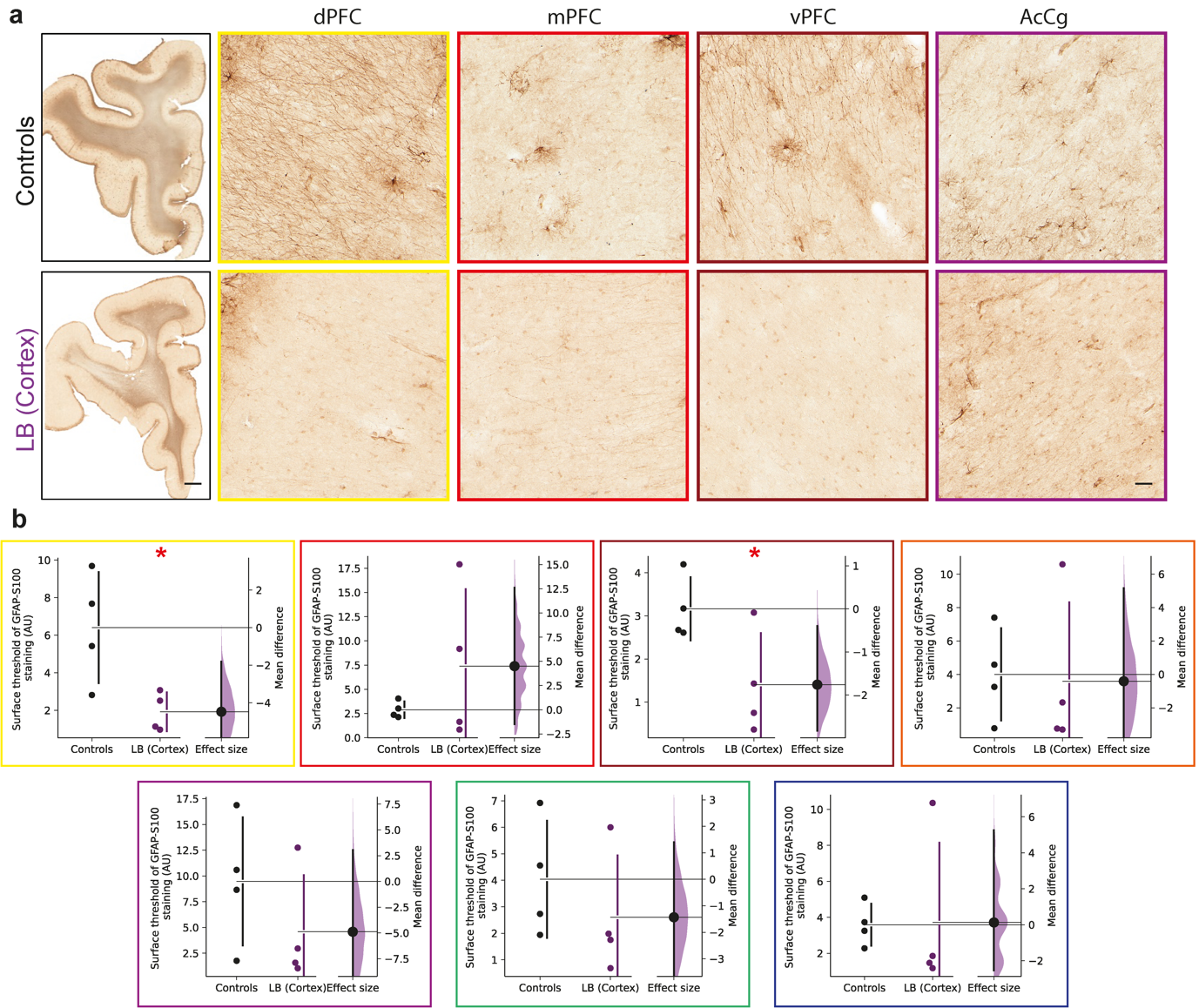


**Supplementary Fig. 4: Astrocytes are decreased in specific cortical regions in non-human primates injected with Lewy Bodies.** Representative images (**a**) and corresponding scatter plots (**b**) of control and LB-injected monkeys with astroglial GFAP-S100 staining in 7 different cortical regions: dorsal prefrontal cortex (dPFC, yellow, p=0.0144, t=2.859), medial prefrontal cortex (mPFC, red, p=0.1515, t=1.126), ventral prefrontal cortex (vPFC, brown, p=0.0232, t=2.503), orbital prefrontal cortex (oPFC, orange, p=0.4427, t=0.1504), anterior cingulate cortex (AcCg, purple, p=0.1418, t=1.177), dorsal premotor cortex (dPMC, green, p=0.2036, t=0.8913), supplementary motor area (SMA, blue, p=0.4792, t=0.05434). Scale bars: top 2mm, bottom 50μm. The horizontal line indicates the average value per group ± SD. The bootstrapped mean difference with 95% CI (error bar) is shown on the right side of each graph. Comparisons were made using unpaired t-tests, *p-value<0.05.


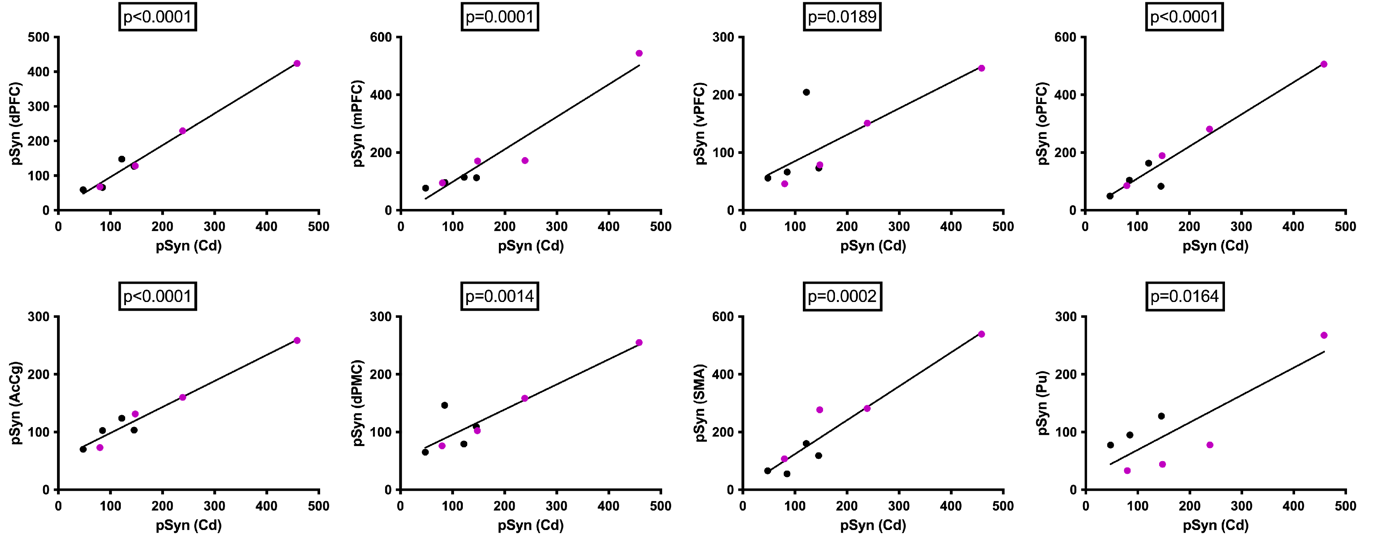


**Supplementary Fig. 5: Phosphorylated** **α-synuclein expression in the prefrontal cortex correlates with the caudate nucleus and the putamen.** Linear regression between phosphorylated α-syn (pSyn) staining using immunoblotting in all 7 cortical regions and the putamen with the caudate nucleus analyzed: dorsal prefrontal cortex (dPFC, p<0.0001, F=342.8, r^2^ = 0.9828), medial prefrontal cortex (mPFC, p=0.0001, F=74.58, r^2^ = 0.9255), ventral prefrontal cortex (vPFC, p=0.0189, F=10.15, r^2^ = 0.6284), orbital prefrontal cortex (oPFC, p<0.0001, F=114.4, r^2^ = 0.9502), anterior cingulate cortex (AcCg, p<0.0001, F=143.9, r^2^ = 0.96), dorsal premotor cortex (dPMC, p=0.0014, F=31.51, r^2^ = 0.8401), supplementary motor area (SMA, blue, p=0.0002, F=64.02, r^2^ = 0.9143), and putamen (p=0.0164, F=12.61, r^2^ = 0.7161). Each dot represents one monkey of the control (black) and LB-injected NHPs (purple).


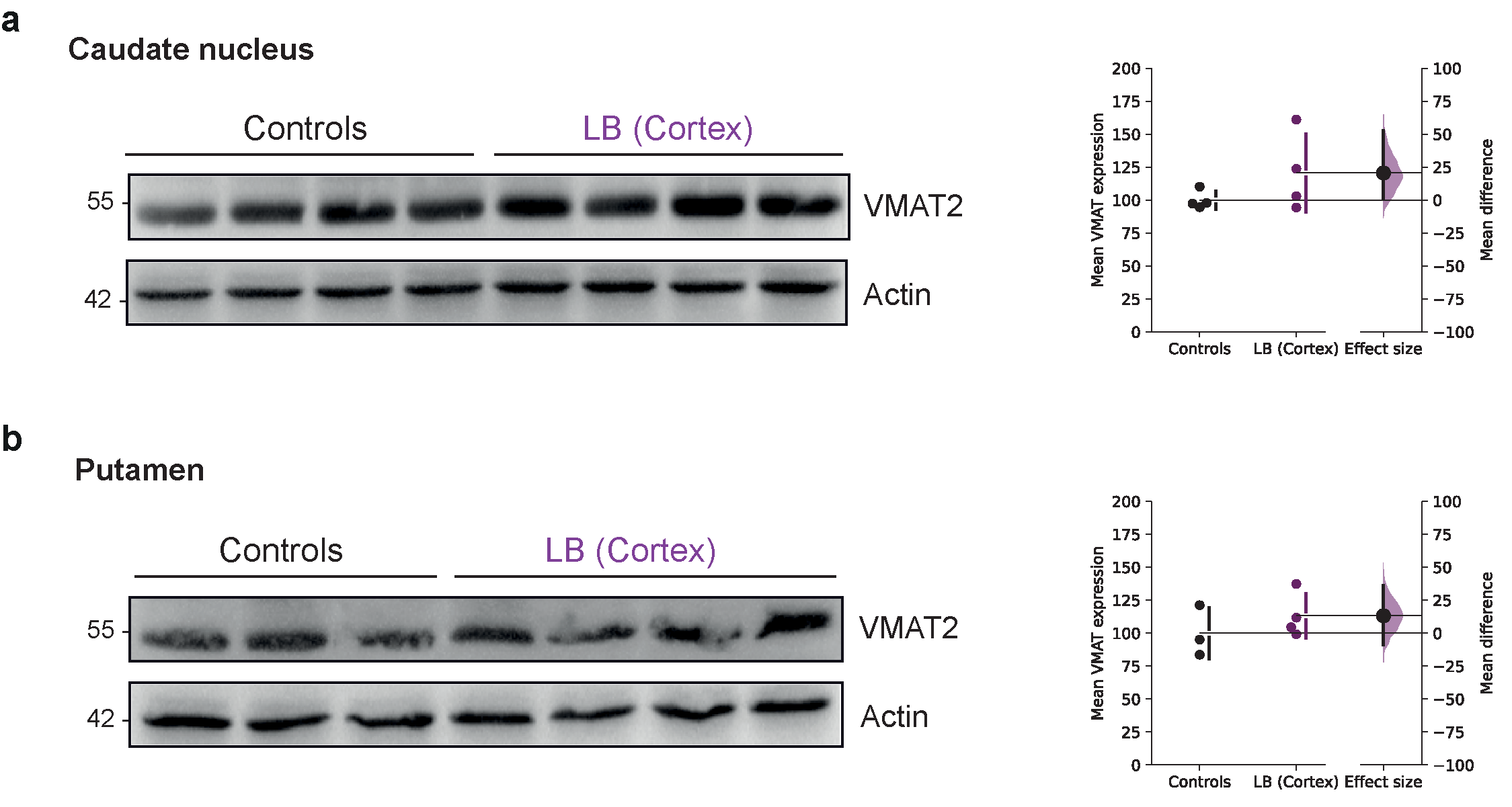


**Supplementary Fig. 6:** **VMAT2 expression remains unchanged in the caudate nucleus and the putamen of LB-injected baboon monkeys. (a-b)** VMAT2 immunoblot levels in the caudate nucleus **(a)** and putamen **(b)** in non-injected and LB-injected baboon monkeys (Caudate VMAT2: p=0.1133, t=1.347; Putamen VMAT2: p=0.1888, t=0.9677). Each dot represents one monkey of the control (black) and LB-injected NHPs (purple). The horizontal line indicates the average value per group ± SD. The bootstrapped mean difference with 95% CI (error bar) is shown on the right side of each graph. Comparisons were made using unpaired t-tests.
