# Supplementary material for: Cortical Lewy body injections induce long-distance pathogenic alterations in the non-human primate brain
Source: NPJ Parkinsons Dis. 2023 Sep 19;9:135. doi: 10.1038/s41531-023-00579-w (PMC10509171; doi:10.1038/s41531-023-00579-w)
Supplement: Supplementary file 3 — Reporting Summary [file 41531_2023_579_MOESM3_ESM.pdf]

nature portfolio

Corresponding author(s): DEHAY Benjamin
NPJPARKD-01998R

Last updated by author(s): Aug 29, 2023

Reporting Summary

Nature Portfolio wishes to improve the reproducibility of the work that we publish. This form provides structure for consistency and transparency in reporting. For further information on Nature Portfolio policies, see our Editorial Policies and the Editorial Policy Checklist.

Statistics

For all statistical analyses, confirm that the following items are present in the figure legend, table legend, main text, or Methods section.

n/a	Confirmed	
☐	☒	The exact sample size (n) for each experimental group/condition, given as a discrete number and unit of measurement
☐	☒	A statement on whether measurements were taken from distinct samples or whether the same sample was measured repeatedly
☐	☒	The statistical test(s) used AND whether they are one- or two-sided<br>*Only common tests should be described solely by name; describe more complex techniques in the Methods section.*
☐	☒	A description of all covariates tested
☐	☒	A description of any assumptions or corrections, such as tests of normality and adjustment for multiple comparisons
☐	☒	A full description of the statistical parameters including central tendency (e.g. means) or other basic estimates (e.g. regression coefficient) AND variation (e.g. standard deviation) or associated estimates of uncertainty (e.g. confidence intervals)
☐	☒	For null hypothesis testing, the test statistic (e.g. F, t, r) with confidence intervals, effect sizes, degrees of freedom and P value noted<br>*Give P values as exact values whenever suitable.*
☒	☐	For Bayesian analysis, information on the choice of priors and Markov chain Monte Carlo settings
☒	☐	For hierarchical and complex designs, identification of the appropriate level for tests and full reporting of outcomes
☒	☐	Estimates of effect sizes (e.g. Cohen's d, Pearson's r), indicating how they were calculated

Our web collection on statistics for biologists contains articles on many of the points above.

Software and code

Policy information about availability of computer code

Data collection	The data supporting the findings of this study are provided in Supplementary Table 1.
Data analysis	Image analysis for microglial morphology was performed in Fiji/ImageJ using custom scripts (available at https://github.com/SoriaFN).<br>GraphPad Prism 10.0 (GraphPad Software, Inc., San Diego, CA)

For manuscripts utilizing custom algorithms or software that are central to the research but not yet described in published literature, software must be made available to editors and reviewers. We strongly encourage code deposition in a community repository (e.g. GitHub). See the Nature Portfolio guidelines for submitting code & software for further information.

Data

Policy information about availability of data

All manuscripts must include a data availability statement. This statement should provide the following information, where applicable:

- Accession codes, unique identifiers, or web links for publicly available datasets
- A description of any restrictions on data availability
- For clinical datasets or third party data, please ensure that the statement adheres to our policy

The data supporting the findings of this study are provided in Supplementary Table 1, and the list of antibodies used in this study is provided in Supplementary Table 2. The data and material supporting the findings of this study are available from the corresponding authors on request.

Research involving human participants, their data, or biological material

Policy information about studies with human participants or human data. See also policy information about sex, gender (identity/presentation), and sexual orientation and race, ethnicity and racism.

Reporting on sex and gender	Age and sex-matched post-mortem samples
Reporting on race, ethnicity, or other socially relevant groupings	*Please specify the socially constructed or socially relevant categorization variable(s) used in your manuscript and explain why they were used. Please note that such variables should not be used as proxies for other socially constructed/relevant variables (for example, race or ethnicity should not be used as a proxy for socioeconomic status).* *Provide clear definitions of the relevant terms used, how they were provided (by the participants/respondents, the researchers, or third parties), and the method(s) used to classify people into the different categories (e.g. self-report, census or administrative data, social media data, etc.)* *Please provide details about how you controlled for confounding variables in your analyses.*
Population characteristics	Fresh frozen postmortem midbrain samples from 5 patients with sporadic PD exhibiting conspicuous LB pathology on neuropathological examination (mean age at death: 67.5 ± 3.5 years; frozen postmortem interval: 17 ± 4h; GIE Neuro-CEB BB-0033-00011).
Recruitment	The samples were obtained from brains collected in a Brain Donation Program of the Brain Bank "GIE NeuroCEB" run by a consortium of Patients Associations: ARSEP (association for research on multiple sclerosis), CSC (cerebellar ataxias), France Alzheimer, and France Parkinson.
Ethics oversight	The consents were signed by the patients themselves or their next kin in their name, following the French Bioethical Laws. The Brain Bank GIE NeuroCEB (Bioresource Research Impact Factor number BB-0033-00011) has been declared at the Ministry of Higher Education and Research and has received approval to distribute samples (agreement AC-2013-1887).

Note that full information on the approval of the study protocol must also be provided in the manuscript.

Field-specific reporting

Please select the one below that is the best fit for your research. If you are not sure, read the appropriate sections before making your selection.

☒ Life sciences ☐ Behavioural & social sciences ☐ Ecological, evolutionary & environmental sciences

For a reference copy of the document with all sections, see nature.com/documents/nr-reporting-summary-flat.pdf

Life sciences study design

All studies must disclose on these points even when the disclosure is negative.

Sample size	For the NHP study, sample sizes of n=4/group were selected as standard for NHP studies to detect large differences betwween treatment and control arms. Group sizes were chosen assuming a one-tailed alpha of 0.05, with sample size of at least three per group, which provided >80% power to detect a difference between the treatment groups and the control group, using a Fisher's exact test.
Data exclusions	No data was excluded from the analysis
Replication	Not applicable. Eight monkeys were used
Randomization	Animals were randomized into treatment or control groups. Four baboons were used for LB intracortical injections and four were untreated control animals.
Blinding	All analyses were performed blinded to the researcher.

Reporting for specific materials, systems and methods

We require information from authors about some types of materials, experimental systems and methods used in many studies. Here, indicate whether each material, system or method listed is relevant to your study. If you are not sure if a list item applies to your research, read the appropriate section before selecting a response.

Materials & experimental systems

n/a	Involved in the study
☐	☒ Antibodies
☒	☐ Eukaryotic cell lines
☒	☐ Palaeontology and archaeology
☐	☒ Animals and other organisms
☒	☐ Clinical data
☒	☐ Dual use research of concern
☒	☐ Plants

Methods

n/a	Involved in the study
☒	☐ ChIP-seq
☒	☐ Flow cytometry
☒	☐ MRI-based neuroimaging

Antibodies

Antibodies used

S129-phopshorylated α-synuclein (pSyn) Elan clone 11A5 Immunohistochemistry 1:5000°
NeuN (Neuronal Nuclei) Merck MAB377 Immunohistochemistry 1:1000°
Dopamine- and cAMP-regulated phosphoprotein- 32 kDa (DARPP-32) Merck MAB4230 Immunohistochemistry 1:500°
Iba1 Abcam ab5076 Immunohistochemistry 1:1000°
Glial Fibrillary Acidic Protein (GFAP) Merck MAB360 Immunohistochemistry 1:2000°
S100 Abcam ab4066 Immunohistochemistry 1:1000°
Tyrosine Hydroxylase (TH) Merck MAB318 Immunohistochemistry 1:5000°
Aromatic L-amino acid decarboxylase (AADC) Merck AB136 Immunohistochemistry 1:1000°
Dopamine Transporter (DAT) Merck MAB369 Immunohistochemistry 1:500°
S129-phopshorylated α-synuclein (pSyn) Abcam ab51253 Biochemistry 1:5000°
α-synuclein ThermoFisher 32-8100 Biochemistry 1:1000°
β-Actin Sigma A5441 Biochemistry 1:10 000°
VMAT2 Abcam AB191121 Biochemistry 1:1 000°

Validation

The antibodies were validated against known standards.

Animals and other research organisms

Policy information about studies involving animals; ARRIVE guidelines recommended for reporting animal research, and Sex and Gender in Research

Laboratory animals

Experiments were conducted as described at the research animal facility of the University of Murcia (Murcia, Spain) 24,25,29. Adult female and male olive baboons (n= 8; Papio papio) ranging from 3 to 14 years of age were housed in two multi-male, multi-female exterior pens. Animals were fed fruits, vegetables, and dry food pellets twice daily before 9 am and after 5 pm. Water was available ad libitum. Allocation to experimental groups was randomized. Four baboons were used for LB injections, and four were untreated control animals.

Wild animals

This study did not involve wild animals

Reporting on sex

Male and Female were used.

Field-collected samples

The study did not involve field-collected samples.

Ethics oversight

Experiments were performed following the European Union directive of September 22, 2010 (2010/63/EU) on protecting animals for scientific purposes. The Institutional Animal Care and Ethical Committee of Murcia University (Spain) approved non-human primate experiments under the license number REGA ES300305440012.

Note that full information on the approval of the study protocol must also be provided in the manuscript.
